# The Importance of Exploring the Role of Anger in People With Psoriasis

**DOI:** 10.2196/33920

**Published:** 2022-03-15

**Authors:** Olivia Hughes, Rachael Hunter

**Affiliations:** 1 School of Psychology Cardiff University Cardiff United Kingdom; 2 Department of Psychology Swansea University Swansea United Kingdom

**Keywords:** psoriasis, skin conditions, psychodermatology, stigma, chronic illness, dermatology, mental health, quality of life

For over 26 years, research has outlined the need for more awareness of the psychological burden of living with a skin condition [1], although the scarcity of research remains an ongoing concern. The All-Party Parliamentary Group on Skin [2] reported that 98% of people in the United Kingdom surveyed were negatively psychologically affected by their skin condition, but only 18% reported receiving psychological support. This discrepancy in care and lack of attention to the role of psychological factors in psoriasis must be addressed if we are to optimize dermatological treatments and patient outcomes. At the very least, the current care pathway could be more psychologically informed to consider the emotional challenges faced by people with psoriasis, providing opportunities for the development of targeted interventions.

There is robust evidence that the clinical course of psoriasis is influenced by social determinants including stress, as well as stressful life events [3], but the exact role emotion plays in the onset and progression of psoriasis seems multifactorial. For example, depression is a common comorbidity in psoriasis, which can be reduced by treatment with biologic drugs, suggesting the potential stigmatizing role of visibility in the psychological impact of the condition [4]. It is perhaps a consequence of the challenges of managing fluctuating skin conditions like psoriasis, including dealing with negative appraisals from other people, that have contributed to reports of anger and aggression among patients [5].

Despite this, the role of anger, whether as an outcome of poor mental health or from stressful life events, remains underexplored. The prevalence of anger is not currently measured within mainstream dermatological services, and considering the potential role of negative emotions in the development, maintenance, and exacerbation of symptoms, exploration could provide valuable insights and benefits for patients. For example, understanding how feeling angry or internalizing aggression could trigger or perpetuate an “itch-scratch cycle” could provide opportunities for intervention [3].

We aim to address this gap in the literature, with a qualitative inquiry to study the complexities of individual experiences and emotions. By developing clearer insights into the role of this emotion, clinicians may be able to better support patients in all aspects of their condition. Specifically, considering psychological contributors and the emotional burden of psoriasis could enable more effective management. For example, combining the physical and psychological manifestations of psoriasis in a holistic approach could promote adaptation, reduce maladaptive coping, and improve patient outcomes. As a minimum, equipping patients with a healthy coping “toolkit” for managing both the physical and psychological effects of psoriasis seems essential.

From a thematic exploration of 12 patient narratives, there appear to be reports suggesting that anger could play a contributory role in the onset and clinical progression of psoriasis for some people. We intend to find answers about how the experience of anger can be addressed to support people living with the skin condition and mitigate potential negative effects. It is time for the 26-year wait to come to an end and for psychological factors to become an integral part of assessment, intervention, support, and research.
